# Experiences of pregnant women exposed to Hurricanes Irma and Maria in the US Virgin Islands: a qualitative study

**DOI:** 10.1186/s12884-022-05232-7

**Published:** 2022-12-17

**Authors:** Noelene K. Jeffers, Deborah Wilson, Hannah Tappis, Desiree Bertrand, Tener Veenema, Nancy Glass

**Affiliations:** 1 Johns Hopkins Bloomberg School of Public Health, Baltimore, MD USA; 2 Johns Hopkins University School of Nursing, Baltimore, MD USA; 3 Jhpiego, MD Baltimore, USA; 4 Augusta University College of Nursing, GA Augusta, USA

**Keywords:** Cyclone, Disaster, Pregnancy, Pregnancy outcome, Resilience, Risk, US Virgin Islands

## Abstract

**Introduction:**

Hurricanes Irma and Maria made landfall in the US Virgin Islands (USVI) in 2017. To date, there is no published literature available on the experiences of pregnant women in the USVI exposed to these hurricanes. Understanding how hurricanes affect pregnant women is key to developing and executing targeted hurricane preparedness and response policies. The purpose of this study was to explore the experiences of pregnancy and birth among women in the USVI exposed to Hurricanes Irma and Maria.

**Methods:**

We employed a qualitative descriptive methodology to guide sampling, data collection, and analysis. Semi-structured interviews of 30–60 min in length were conducted with a purposive sample of women (*N* = 18) in the USVI who were pregnant during or became pregnant within two months after the hurricanes. Interviews were transcribed verbatim and data managed in MAXQDA. Team members developed a codebook, applied codes for content, and reconciled discrepancies. We thematically categorized text according to a socioecological conceptual framework of risk and resilience for maternal-neonatal health following hurricane exposure.

**Results:**

Women’s experiences were organized into two main categories (risk and resilience). We identified the following themes related to risk at 3 socioecological levels including: (1) individual: changes in food access *(We had to go without)* and stress *(I was supposed to be relaxing); (2)* household/community: diminished psychosocial support *(Everyone was dealing with their own things) and* the presence of physical/environmental hazards (*I was really scared*); and *(3)* maternity system: compromised care capacity *(The hospital was condemned).* The themes related to resilience included: (1) individual: personal coping strategies *(Being calm)*; (2) *household/community*: mutual psychosocial and tangible support *(We shared our resources);* and (3) the maternity system: continuity of high-quality care *(On top of their game)*.

**Conclusions:**

A socioecological approach provides a useful framework to understand how risk and resilience influence the experience of maternal hurricane exposure. As the frequency of the most intense hurricanes is expected to increase, clinicians, governments, and health systems should work collaboratively to implement hurricane preparedness and response plans that address pregnant women’s unique needs and promote optimal maternal-infant health.

**Supplementary Information:**

The online version contains supplementary material available at 10.1186/s12884-022-05232-7.

## Introduction

Hurricanes Irma and Maria made landfall in the US Virgin Islands (USVI) on September 6, and September 20, 2017, respectively. Hurricane Irma caused widespread destruction, predominantly on St. Thomas and St. John, whereas Hurricane Maria devastated the island of St. Croix. As of March 2019, the joint estimated economic impact totaled approximately $1.54 billion along with acute stressors to public and private healthcare systems and processes, the full economic and human toll of which as yet remains unknown [1].

Combined, the hurricanes cut the daily inpatient capacity of the two major hospitals on the islands in half. Both hospitals sustained immense infrastructural damage and a combined attrition of approximately 138 medical staff members [1]. Some healthcare providers in private practice were unable to immediately resume clinical services, and others eventually left the territory due to damage to their homes or their medical offices [2, 3]. The impact of the hurricanes to routine and emergent inpatient and ambulatory care was significant; however, specific details regarding the hurricanes’ impact on the maternity health system and the care of pregnant women have not been published.

Pregnant women exposed to hurricanes are at risk for adverse maternal and neonatal outcomes [4]. As the most severe hurricanes are projected to grow in severity and frequency due to climate change [5], understanding these risks becomes increasingly important. Studies of hurricanes in the United States and Australia have shown that exposure to a hurricane is associated with no, late or inadequate prenatal care [6, 7], preterm birth [8–10], and increased rates of cesarean Sects. [6, 11, 12]. Hurricane exposure and the associated destruction appear to negatively impact the fetus and neonate by increasing the risk of fetal death [13, 14] and neonatal morbidity [11]. The pathways through which hurricane exposure increases maternal and neonatal health risks are not completely understood. Severity of exposure and the associated stress accompanying traumatic events may explain why some pregnant women avoid harm while others are negatively impacted. Severe hurricane experiences, such as walking through flood waters, being injured, or seeing a loved one die, are associated with increased low birth weight [15]. Those that experienced three or more severe hurricane experiences had significantly greater odds of postpartum post-traumatic stress and depression symptoms [16].

While in-depth descriptions of women’s experiences of pregnancy and birth during and after hurricanes can provide insight into how those individual experiences shape maternal and neonatal health outcomes, few published studies exploring these experiences exist. In two such studies, women who were pregnant during Hurricane Katrina in Louisiana or Typhoon Haiyan (Yolanda) in Japan described the stress associated with maintaining regular prenatal care, ensuring the wellbeing of their baby, and accessing diminished maternity services [17, 18]. Participants reported struggling with depression and post-traumatic stress while striving to meet their own needs and those of their family members [17, 18]. After Hurricane Katrina, women coped with the disruptions to their lives by creating new support networks [17]. The impact of hurricanes on public and private infrastructure can persist beyond the first few months of recovery. In one study exploring the experiences of Puerto Rican women who were pregnant during Hurricane Maria and its aftermath, almost a quarter of participants reported that they were still displaced from their homes one year after the Hurricane [19]. Additionally, 5 to 7 years after Hurricane Katrina, women were still experiencing significant disruptions in housing, employment, and psychosocial support, which negatively impacted their mental health [20]. These extant studies provide important information about the impact of hurricanes on the experience of pregnancy after hurricanes; however, they do not provide in-depth descriptions within a framework of risk and resilience. Additionally, to date, there are no published studies focusing on the experience of women in the USVI who were pregnant during and immediately after Hurricanes Irma and Maria.

To address this gap in knowledge, the purpose of this qualitative study is to describe the pregnancy and birth experiences of women in the USVI following Hurricanes Irma and Maria. Through in-depth interviews we identified factors that contributed to maternal and neonatal health risk and resilience.

## Methods

### Study participants

We employed the Consolidated Criteria for Reporting Qualitative Research (COREQ) checklist in reporting the findings [21]. The purposive study sample included 18 women. Participants who met the following criteria were originally included in the study: (1) were 18 years of age or older; (2) were in the USVI during Hurricane Irma, Maria, or both; (3) were pregnant during the Hurricanes; and (4) gave birth in the USVI. We expanded the inclusion criteria after initial recruiting efforts, in order to increase the number of participants. The expanded inclusion criteria also included women who became pregnant within two months after the Hurricanes and those who gave birth off island.

The recruiting team comprised three researchers who identified as women: the first author, a Certified Nurse Midwife and PhD candidate trained in qualitative research methods, and two trained local research assistants. Both research assistants held graduate degrees and were experienced in research study recruiting methods. Recruitment strategies included word of mouth, Facebook posts in groups for residents of the USVI, snowball sampling, flyer distribution in public places, including daycares and medical offices, and events. We recruited participants with maximum possible variation with respect to age, parity, socioeconomic status, and perinatal outcomes to gather a wide variety of experiences [22]. None of the participants had any prior relationship with the interviewer. One participant did not attend her scheduled interview after completing the initial consent process and did not call us prior to the interview to reschedule. The research team made four attempts to contact the participant to reschedule the interview without success. Recruitment ceased once saturation was achieved and it was determined that additional interviews would not elicit additional unique information pertinent to the research questions [23, 24].

### Data collection

The first author conducted interviews by phone between July 2019 and September 2019. The interviewer read the participants information about the purpose of the research study, the risks and benefits of participation. We informed participants that the research study was part of the doctoral dissertation by the first author, who was a former resident of the USVI. Each participant provided oral consent. The interviewer compensated participants with a $25 gift card. The interviews lasted 30–60 min and were audio-recorded with participant consent. Table 1 displays the full interview guide, which included open-ended questions designed to explore participant experiences shortly before, during, and after the hurricanes. The interviewer used probing questions to explore details of the hurricane preparation and recovery and understand the impact of the hurricane. Three pilot interviews were initially conducted and the first author made minor edits to the interview guide following those pilot interviews. Ethics approval for the interviews was provided by the Johns Hopkins University School of Medicine Institutional Review Board (IRB#: 00212679).

**Table 1:** Interview guide

• Before Hurricanes Irma/Maria, what were your expectations for this pregnancy?	
• In the days leading up to the hurricanes, what sort of concerns did you have about your pregnancy?	
• When thinking about concern for your pregnancy and other potential concerns, such as safety for your family, how did you determine what to prioritize?	
• Tell me about the night that Hurricane Irma/Maria hit. What was that like for you?	
• In the days and weeks after the Hurricane, what was it like to get the care that you needed for your pregnancy?	
• What actions did you take to make sure you received adequate care for the pregnancy?	
• What made it hard for you, as a pregnant woman, to get through the hurricane?	
• What complications did you experience during your pregnancy and birth?	
• What was it like to manage your pregnancy along with your other concerns?	
• What impact, if any, did the Hurricanes have on your pregnancy/general health?	
• What were some of the resources you used to help you meet your needs?	
• How were your expectations for your pregnancy different from what you experienced?	
• What were some of the differences you experienced between your prior pregnancies and this pregnancy? (If multiparous)	
• What impact do you think that living in a U.S. territory may have had on your experience?	
• What might help other pregnant individuals in the future to successfully cope with the changes that come along with dealing with a severe hurricane while pregnant?	
• What resources should be available to pregnant women during severe hurricanes?	
• Is there anything else you feel is important to tell us about your experience with pregnancy and birth during and after the hurricanes?	

### Reflexivity

Throughout the research process, the first author practiced reflexivity [25]. She was aware that her personal experience of growing up in the USVI, experiencing Hurricane Hugo, a destructive Category 5 hurricane that made landfall in the USVI in 1989, and subsequently conducting research as a current resident of the US mainland might impact the data collection and analyses processes. Specific actions included informal self-reflexive processes of how her identity as an insider-outsider might influence the development of an unbiased interview guide, decisions by participants about whether to take part in the study, and the interpretation of the findings. She also engaged in reflexive practices with a senior team member in which she identified and confronted assumptions during the data collection and analysis process.

### Analysis

#### Conceptually-informed analytic strategy

**Fig. 1:**
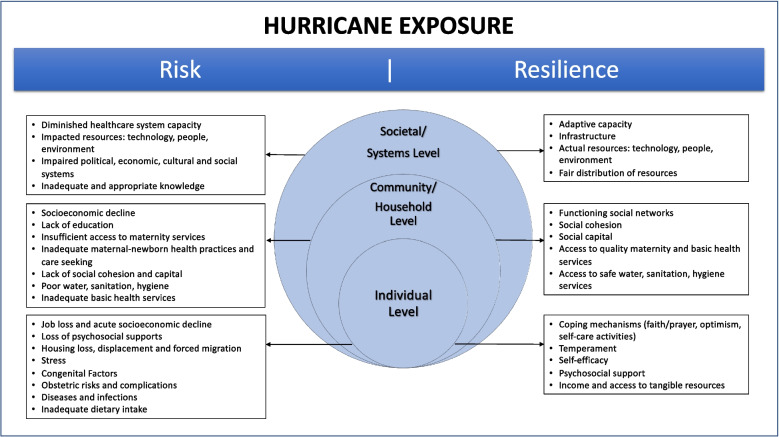
Maternal-neonatal health, risk and resilience following hurricane exposure conceptual framework. (adapted from the UNICEF Conceptual Framework for Maternal and Neonatal Morbidity and Mortality) [26]

A framework of risk and resilience provides a useful lens through which researchers, health providers and policy makers can understand the impacts of maternal hurricane exposure on pregnancy, birth, and postpartum. We derived an adapted framework for our study from the UNICEF Conceptual Framework for Maternal and Neonatal Morbidity and Mortality which identifies the causes of maternal and neonatal deaths [26]. The adapted framework outlined in Fig. 1 proposes that factors of risk and resilience influence maternal and neonatal health following hurricane exposure. The framework proposes that risk and resilience arise from a variety of interrelated settings based on Bronfenbrenner’s ecological systems theory [27], starting at the individual level and extending outward to incorporate the household/community, and structural levels. In this framework, we identified risks as contributors to maternal and neonatal morbidity and mortality. Risks associated with hurricane exposure include factors such as diseases and infections, poor water and hygiene, inadequate dietary intake, and impaired economic, cultural and social systems. The framework also incorporates resilience, defined as the capacity “to adapt successfully to disturbances that threaten the viability, the function, or the development of that system” [28]. Components of resilience might include protective factors such as psychosocial support, social capital, and the existence of resources including technology, people or infrastructure. We used this adapted framework to develop a semi-structured interview guide and analyze the interview transcripts.

#### Analytic procedures

Each interview was professionally transcribed, checked for accuracy, and imported into MAXQDA 2020 for data management [29]. The first author created an initial codebook with a priori codes prior to data collection, guided by a literature review and the conceptual framework [30]. Two authors (NJ and DW) independently read each transcript. Transcripts were not returned to the participant for comments. Consistent with a qualitative descriptive methodology, we coded each transcript based on content, summarizing the events and experiences detailed by the participants [31]. During the coding process, authors NJ and DW made minor changes to the initial codebook (see Supplementary File 1). Coding discrepancies were negotiated and resolved through discussion [31]. We organized the coded data into categories according to the framework. We grouped participant experiences into the three ecological levels identified in the conceptual framework, and further analyzed the interview transcripts for how they contributed to the women’s experiences of risk and resilience during and after the hurricanes. Themes were identified through a deductive process guided by the conceptual framework. Data from the interviews were organized within three levels adapted from Bronfenbrenner’s ecological systems theory: individual, household/community, and society/system [27]. Sources of both risk and resilience for hurricane-affected pregnant women arise from each interrelated level.

## Results

### Participant characteristics

Interview participants (see Table 2) were mostly African Caribbean/Black (*n* = 11). Three participants self-identified as multiracial, while the remainder (*n* = 4) were white. Three participants reported their ethnicity as Hispanic/Latina. The majority had a high school level of education or less (*n* = 10) and were employed full-time (*n* = 12). Annual household income varied; 6 participants reported incomes less than $25,000 per year, 7 reported incomes between $25,000 and $60,000, and 5 reported incomes over $60,000 per year. Ten of the 18 participants were married. The average age of participants was 31 years (overall age range between 18 and 42 years). At the time of the hurricanes, participants lived on St. Thomas (*n* = 9), St. Croix (*n* = 8), and St. John (*n* = 1). Fourteen women gave birth in the USVI, while 4 gave birth on the US mainland. All participants gave birth at a hospital. Over half (*n* = 10) of the participants reported that they experienced high-risk pregnancies with conditions including multiple gestation, hypertension, or gestational diabetes.

**Table 2:** Participant sociodemographic, pregnancy, and birth characteristics

Characteristic	n (%)
**Age**	
18–24	2 (11.1%)
25–29	6 (33.3)
30–34	7 (38.9%)
35–39	1 (5.6%)
40–44	2 (11.1%)
**Race**	
Black/African Caribbean	11 (61.1%)
White	4 (22.2%)
Multiracial	3 (16.7%)
**Ethnicity**	
Hispanic	3 (16.7%)
Not Hispanic	15 (83.3%)
**Marital Status**	
Never Married	9 (50.0%)
Married	8 (44.4%)
Divorced	1 (5.6%)
**Employment**	
Not Employed	5 (27.8%)
Part-Time	1 (5.6%)
Full-Time	12 (66.7%)
**Education**	
Less than High School	5 (27.8%)
High School	5 (27.8%)
Some College	3 (16.7%)
College	2 (11.1%)
Graduate	2 (11.1%)
Post-Graduate	1 (5.6%)
**Household Annual Income**	
$0-$15,000	3 (16.7%)
$15,000-$25,000	3 (16.7%)
$25,000-$60,000	7 (38.9%)
Greater than $60,000	5 (27.8%)
**Island of Residence**	
St. Croix (Hurricane Maria)	9 (50.0%)
St. Thomas (Hurricane Irma)	8 (44.4%)
St. John (Hurricane Irma)	1 (5.6%)
**Gave Birth in the USVI**	
Yes	14 (77.8%)
No	4 (22.2%)
**Type of Birth**	
Vaginal	9 (50%)
Cesarean	9 (50%)
**Timing of Birth**	
Full Term	18 (100%)
Pre-Term	0 (0%)
**Hypertension**	
Yes	5 (16.7%)
No	13 (83.3%)
**Delay or Interruption in Prenatal Care**	
Yes	12 (66.7)
No	6 (33.3)

### Themes

We analyzed narratives of hurricane and hurricane recovery experiences within three socioecological levels: the individual, household/community, and maternity system levels. Five major themes of risk emerged at the individual level (changes in food access, stress), the household/community level (diminished psychosocial support, the presence of physical/environmental hazards), and the maternity system level (compromised care capacity). Four major themes of resilience arose at the individual (personal coping strategies), household/community level (mutual psychosocial and tangible support), and maternity system level (continuity of high-quality care) emerged. Each major theme is described below with exemplar quotes from participants.

### Individual

#### Risk

##### Changes in Food Access (“We had to go without”)

Participants described substantial changes to their access to fresh and nutritious foods after the hurricane. For many participants, pre-hurricane preparations included the anticipation of loss of electricity for an extended period, thus precluding the ability to safely refrigerate fresh food. Some procured large supplies of canned foods and bottled water. Others reported more modest preparation efforts because they did not expect the hurricane to be severe. However, after the hurricanes, access to grocery stores was limited and non-perishable food items did not last long. Roads were blocked by fallen trees and debris. Some stores had been heavily damaged and thus remained closed or offered limited supplies. Participants reported being limited to stores within walking distance. Access to prenatal vitamins was disrupted when patients were unable to access pharmacies in a timely manner. Additionally, the curfew imposed immediately after the hurricanes restricted people to a 4-hour window during which to travel on the islands, exacerbating the difficulty of gathering necessary supplies. Participants recalled relying on public distribution of meals, ready-to-eat (MREs) as a main source of food. Long lines and rationing at grocery stores further frustrated participants. Given the significant changes in nutrition, participants reported concern that their baby was not receiving enough nutrients. One participant attributed her low gestational weight gain to the inadequate food availability:

*And I lost a lot of weight… a lot. I wasn’t gaining– I gained - I kid you not - nine pounds through my whole entire pregnancy…The food was horrible, canned food. I wasn’t eating healthy…The grocery store didn’t have fresh food as much.*

##### Stress (“I was supposed to be relaxing”)

Participants frequently cited stress as a major concern. Common stressors included contending with lack of electricity, unstable food and water, changing diets, alterations to employment, and fluctuations in income. One participant described the difficulty she experienced confronting these stressors combined with pregnancy.

*A hurricane is not an ideal situation and– you know, for anybody to be in. But it becomes more complicated when you’re pregnant. And the stress that it puts on you is a lot… a lot of people probably wouldn’t be able to handle some of the stuff that I went through…with no lights and no running water…while you’re pregnant.*

Participants frequently asserted that stress had negative effects on their pregnancy experience, and with how the stress would impact their baby.

*I tried my best not to stress, but I was so worried that there was going to be something wrong with her because... I’m supposed to be relaxing.*

Although friends and family offered shelter and other resources, displacement was a source of significant stress. One participant described the negative emotional toll of the early postpartum period caused by enduring displacement while transitioning into her role as a new mother.

*Even though it was a blessing getting everything that we needed from my in-laws, it was also really– I won’t lie. Just dramatic. And it was like, “Can’t I be a mom by myself? Can I just be in my own privacy? Do I have to live with someone else?“ Emotionally, it was…a lot for me in terms of just not being able to have my own privacy with my daughter as a new mom.*

Several women also described lingering symptoms consistent with depression and post-traumatic stress disorder (PTSD) after the hurricanes. Some reported experiencing significant fear and anguish with impending thunderstorms or milder hurricanes after their experiences with Hurricanes Irma and Maria.

#### Resilience

##### Personal coping strategies (“Being Calm”)

Participants reported using various coping mechanisms to deal with hurricane-related stress. For some, prayer and religious faith were essential. Positive thinking, visualization, and gratitude were also employed. Many participants actively worked to maintain calm due to concern about the effect stress could have on their pregnancy.

*I really did not want to have a home birth. That’s why I was really so calm in my head and just trying not to have the pressure of the hurricane induce my pregnancy.*

Several participants coped by not focusing too intently on their situation. Rather than delving into feelings of loss, some women focused on survival and post-hurricane recovery, preparing for their birth, or on meeting the needs of other family members. One participant described how she was not able to fully process the complex emotions surrounding the trauma of the situation at the time of the recovery. Rather, the ability to process came later, after the acute recovery period had passed:

*I didn’t get to mourn or go through the process of “we’re going through this aftermath.” I feel like the entire time I was pregnant, I was just focused... on being calm the entire time. I didn’t get to like take in [that] “this is devastating.” It was not after maybe like months after, was when I kind of like broke down and cried and realized we went through a category five hurricane and I was very pregnant and I had my 18-month old.*

### Household and Community

#### Risk

##### Diminished psychosocial support (“Everyone was dealing with their own things”)

Coping with the hurricane recovery period was especially difficult for those with little support from friends and partners. Participants reported that receiving help from friends and family was sometimes difficult because they were managing their own needs:

*Everyone was dealing with their own things and dealing with their houses and things like that. Typically…my mom would have come or my mother-in-law or my sisters in law, but everyone was dealing with their own things at that time. I mean they would still check up on me, but it wasn’t like how it would have normally been.*

A minority of participants (*n* = 2), noted that lack of support from employers negatively impacted their ability to prepare to meet their needs during and after the hurricane. One participant noted that her employer made it difficult for her and her colleagues to prepare for the hurricane.

*They didn’t let us [leave work to get sandbags]. I [contacted HR]. I emailed everybody because I was so upset, but it just got ignored, conveniently…Their idea was, “Well, you’re here to serve the public...“ I’m like, “Well, that’s perfect, but who’s here to help out the people who serve the public?“ Who’s here to give us our little opportunity to prepare our homes? And that was an ongoing complaint between the administration and the [employees].*

##### Physical/Environmental hazards (“I was really scared”)

Participants described managing pregnancy in unsafe or dangerous circumstances during and after the hurricanes. During the hurricane, several women reported having to bend repetitively to mop flood waters that entered their homes. One participant recounted that she fell and slipped on pooled hurricane water but this participant did not have the ability to safely seek medical attention during the hurricane. One woman experiencing regular uterine contractions on the night of the hurricane, (her due date), recounts how she and her parents waded through flood waters to find help.

*The water was waist deep to me…The water was just so high and dark, and I was scared. I was really scared. So, my parents cupped my hands between theirs… so nobody could trail away, because the current of the water was flowing pretty heavy as well. So, we walked through the water, and I remember stopping a few times because ... not knowing it was contractions ... And, my mom said, “We’re almost there. We’re almost there, baby.“*

Several women reported having heard that the low pressure of hurricane systems sometimes caused women to go into labor. One participant described being nervous that her baby would come early:

*Yeah, we were pregnant, and people have all manner of, “Oh, this is gonna happen, and this is gonna happen.“ And, and so, you know, a lot of people were like, “Oh, yeah premature babies.“ And I’m looking at people like, “No…We’re not having a baby in the middle of a hurricane,“ you know? So, I’m like, rubbing my belly and talking to the baby. I’m like, “You better stay in there.“*

In the days after the hurricanes, driving or walking along the roads was dangerous because of fallen power lines, debris, and downed trees. One woman described climbing over downed trees while pregnant so that she could visit her family. Some participants were located in remote areas of the island without cell or landline phone service and were concerned that they would be unable to call for help in the case of an emergency.

### Resilience

#### Mutual psychosocial and tangible support (“We [shared] our resources”)

Although participants reported that they received inconsistent or minimal support from family and friends, most participants, however, described having abundant support from friends, family, coworkers, or church members. Neighbors checked on each other, sharing food and valuable information. Many participants had friends and family nearby who provided encouragement and tangible assistance:

*My family and friends…they helped me a lot, especially my family [be]cause they came to check up on me to make sure that if we didn’t have anything we would be able to get it. My friends helped me out as well because if somebody was cooking… they would give everybody a place to eat…And we would share our resources of food, [and] water…They was also making sure that, you know, I was safe, and I was okay.*

One woman described how she stopped caring for herself—for example, she stopped combing her hair—because of the stress, and how family came to her aid:

*They were a very big support. I was so stressed– I was [not] really combing my hair…. And my aunt was like, like, “Hey, we need to do something at least before you go into the hospital.“ So, she would braid it up for me. So, they were a very big support for me.*

Employed participants noted that their employers were a source of significant help. Some participants continued to receive pay even though their place of work was temporarily closed due to damage. Others noted that their workplaces provided them with supplies e.g. generators, fans, food, and water.

*And thank God I had such a great boss that, you know, she was very lenient. If I couldn’t come in on certain days…she wouldn’t expect me to come in. I can’t complain…And not to mention as far as my employer, she was pretty helpful, like, “Okay, do you guys need water? Do you guys need this?“ So that was a big help as well too.*

Some participants talked about the benefits of living in an area that has experience with recurrent cycles of hurricane exposure and recovery. Having endured destructive Category 4 and 5 hurricanes before, they noted that the recovery process improved with each hurricane.

*If [we were in] in any other country or island, I don’t think we would have been recovering so quickly. And we did have a really good recovery turnover after Maria…I’ve been through Hugo, and, you know, [this time] things wasn’t that bad, you know?*

### Maternity system level

#### Risk

##### Compromised care capacity (“I heard that the hospital was condemned”)

Participants recalled concern regarding how the hurricanes’ effects on hospital infrastructure and resources would impact their birth. Many women contemplated leaving the USVI for their birth. Participants reported that they heard that much of the hospital had received major damage, that it was partially condemned, that there was only one functioning operating room, and that some services were being provided in mobile tents. However, despite these rumored deficiencies, some were too close to their estimated due date to travel off island, either before or after the hurricanes. Others did not have the financial resources necessary to leave even if they would have wanted to give birth elsewhere. Participants with lower risk pregnancies noted that their healthcare providers reassured them that the hospital’s labor and birth services were suitable.

Most participants (*n* = 14) made an intentional decision to stay in the USVI for birth and found that the labor and birth floor had been largely spared and was functional. However, it still lacked many of the comforts and conveniences present prior to the hurricanes.

*There was no hot water in the hospital... The hospital was technically condemned, and so that was a huge stressor for me, but the maternity ward and postnatal ward was supposed to be of good condition...but that was extremely stressful. I remember I really freaked out when I had heard that the hospital was condemned, but I didn’t want to leave island because I didn’t want to be away from my significant other for his first child. I didn’t want that opportunity to be lost. They didn’t tell you that they didn’t have warm water either, so I went in for a shower. I ended up taking a cold, cold shower post C-section and oh my God.*

One participant noted that she overheard staff lamenting that they were in the hospital for days after the Hurricane without being able to attend to their own families and homes. Another attributed her cesarean to the altered hospital capacity and infrastructure. Some participants experiencing high risk pregnancies (*n* = 4) noted that their healthcare providers recommended that they leave the island. Three of them chose to leave the island and 1 remained. This participant, carrying twins, was originally planning to remain on the island for her birth. However, after the hurricane hit, she recounts how her healthcare provider assisted her with leaving the island for her birth.

*She kept trying to get us on a mercy flight…My pregnancy was already high-risk because I was carrying twins…They wanted to get the people with more serious problems out based off of how much was damaged, pregnant people and people with other problems… At the time they didn’t have the necessary materials in case my babies were born [early].”*

Most participants reported having an interruption in their prenatal care ranging between 2 and 4 weeks. Many had a difficult time contacting their health care provider’s office, complicating their attempts to resume prenatal care after the hurricanes. Some medical offices were damaged and physicians utilized temporary locations. Landline phones did not work, making attempts to contact health care providers still more difficult.

*Um, it was a delay…I would say about for maybe like 2 to 4 weeks…But their building had got ruined, so they had to move somewhere else. And where they moved [the space was] a lot smaller. It took a little time for them to get over there. But I would say three weeks…*

This participant was offered a non-emergent, elective cesarean section when she went past her due date, but declined. With the impending hurricane, she made last-minute preparations for a potential unplanned, undesired, and unassisted homebirth should she go into labor during the hurricane.

*They told [my partner] what to look for and he had ... two older kids [born through homebirth] already, so he knew just in case if I were to have a baby during the hurricane this is what to do. [My family was] prepared in regards to that, and luckily, we didn’t need that… I mean I was thankful that we were prepared, but I really did not want to have a home birth.*

### Resilience

#### Continuity of high-quality care (“On top of their game”)

Healthcare professionals and the hospital collaborated to ensure that women received quality maternity care. One participant who was past her due date during Hurricane Maria noted that a Certified Nurse Midwife on the island came to her house to ensure her wellbeing.

*That day, my boss’s husband, who was a federal agent at the time, and his friend went up to the north shore road to see if I was okay. Then, I was fine. Then after that, another [midwife], came by the house to see if I was okay. Everyone was worried because I was 42 weeks and I was still not having any contractions or anything like that.*

Many participants recounted that their obstetric care providers’ offices were destroyed and that they were forced to receive care elsewhere. One participant noted that her provider started seeing patients in the hospital and that, because of a cloud-based electronic health records system, her provider was able to access her medical records.

*The office was destroyed…she had to move to a temporary location where she was still seeing her patients at the hospital… So, my prenatal visits were just as frequent or regular, like, if I were– not had the hurricane because she didn’t make it any– um, it’s just that it was not in her office because it was destroyed... And most of her information was, you know, something online. So, she could have used her laptop to access our records. So that was a plus.*

Some participants visited their provider’s office location in person to make an appointment. Others reported using social media, such as Facebook, or apps like WhatsApp to locate a cell phone number for their provider.

*So about a week after the hurricane, we had no power or Internet, but in town, there was internet… A lot of the offices would post on Facebook temporary phone numbers. So, I think [the doctor’s] office actually posted on Facebook a cell phone number.*

Despite pervasive concerns regarding the hospital’s ability to provide adequate labor and birth care, 14 of 18 participants gave birth in the USVI. All but 1 of the participants who gave birth in the USVI reported overall positive experiences with the care, the staff, and the facilities. Participants reported an adequate amount of staff and only minor damage to the labor and delivery floor. For example, one participant noted that, despite the lack of conveniences like hot water for baths, the quality of the care was good:

*…The nurses and everyone there were amazing*…*My husband would tell me they didn’t have running water, and they had to, like, boil water to bring it for his first bath. And, I [didn’t] remember, until he told me, because they were just that good. They...really were on top of their game.*

## Discussion

The purpose of this study was to explore the experiences of pregnancy and birth among women in the USVI exposed to Hurricanes Irma and Maria and to include in-depth descriptions of risk and resilience. The findings demonstrate that hurricanes and the recovery environment pose a number of risks to pregnant women’s nutrition, physical safety, and mental health. The hurricanes’ destructive effects on ambulatory and inpatient processes and infrastructure further imperil high quality care. To cope with these risks, women draw on multiple sources of resilience, including their own coping mechanisms and the support and care from their support networks. Women also rely heavily on the maternity system’s ability to respond to the hurricane’s disastrous effects and maintain continuity of care to help ensure a safe pregnancy and birth.

### Risk and resilience at the individual level

Women in this study discussed significant changes in their nutrition and access to prenatal vitamins after the hurricanes. Difficulties in sourcing food and going without food has previously been described within the context of hurricane evacuation [17, 18]. None of the women in our study were evacuated but many experienced difficulties in securing food while in their own homes or while temporarily displaced. Many expressed concern that these dietary changes would negatively impact fetal development. Their concerns are consistent with evidence that post-hurricane changes in nutrition quality and access has been linked to preventable birth defects. For example, when Hurricane Gilbert made landfall in Jamaica in 1988, the island experienced a significant rise in neural tube birth defects related to changes in access to foods high in folic acid [32]. Given challenges in accessing food stores and pharmacies immediately after hurricanes and concerns around rationing, it is essential to provide adequate public health messaging and anticipatory guidance to pregnant women regarding pre-hurricane food stockpiling, maintaining adequate nutrient and folic acid intake, and the prevention of neural tube and other preventable birth defects.

Participants identified hurricane-related stress as a major challenge during pregnancy and postpartum. Some of these narratives mirror narratives of stress analyzed in prior research [17, 18]. The women in our study revealed that they were concerned about the impact of stress on the fetus and on their transition to parenthood. Anticipating hurricane-related stress and providing key mental health supports is critical because stress following maternal hurricane exposure has been linked to preterm birth [8, 9, 15], postpartum depressive and post-traumatic stress symptoms [16]. Although the perspectives shared in this study primarily focused on stress in the immediate aftermath of the hurricanes, there is evidence that maternal hurricane exposure is associated with stress, depression, and post-traumatic stress symptoms extending as many as 5 to 7 years after hurricanes [20]. Therefore, short- and long-term mental health support are necessary to help women successfully navigate two simultaneous life stressors: childbearing and hurricane recovery.

Participants reported employing various self-directed coping strategies. Participants frequently cited positive thinking, staying calm, and a reliance on faith as factors that contributed to their individual resilience. Similarly, Hurricane Katrina survivors depended on faith, religion, and spirituality to cope [33, 34]. Helping women strengthen pre-existing and develop new coping strategies may help promote their recovery and contribute to resilience.

### Risk and resilience at the household/community Level

Pregnant women are often exposed to dangerous circumstances and hazards after hurricanes. Evidence suggests that experiencing dangerous circumstances after hurricanes may put them additional risk of experiencing adverse pregnancy outcomes. For example, researchers in one prospective study conducted after Hurricane Katrina found that women who experienced injury during the hurricane were at increased risk for preterm birth [35]. Counseling women to identify a safe place to stay and avoid slips, falls, and other dangerous circumstances during and after a hurricane is an important point of pre-hurricane guidance that could potentially mitigate adverse pregnancy outcomes.

Some participants experienced a withdrawal of support at the household/community level, potentially further compounding the negative mental health effects of their hurricane experiences. Family and friends, preoccupied with employment or their own hurricane recovery efforts and responsibilities, were not always able to meet the participants’ psychosocial and tangible needs in the same way as they might have before the hurricane. Lack of social support among pregnant women after Hurricane Katrina has been linked to depressive and PTSD symptoms and perceived stress [20]. However, most participants noted examples of spontaneous community coordination in which family, friends, and neighbors shared food, resources, and information. These spontaneous behaviors describe a form of social capital, which has been recognized as a component of community resilience [36]. Social capital, which incorporates bonding, bridging, and linking between social networks, has been associated with how communities successfully adapt and recover after natural disasters [37, 38]. In Puerto Rico, social capital as expressed through communal cooking, cooperative debris removal, and community food donations contributed to recovery and resilience after Hurricane Maria [39]. Future interventions to prepare and respond to hurricanes should highlight efforts to develop and cultivate social capital to maintain and even increase the social support pregnant women receive during and after the crisis to improve their mental health trajectory.

### Risk and resilience at the maternity system Level

The interviews revealed that women contended with minimal pre-hurricane anticipatory guidance, delayed prenatal care, displaced maternity care providers, inaccessible medical records, and damaged hospital facilities. However, narratives also revealed resilient practices like the use of social media in the post-disaster setting, cooperation between private healthcare providers and hospitals, home visits, and coordinated care planning for high risk pregnancies. The maternity system, although severely affected, exhibited an ability to absorb disruptions and respond to them effectively, in some cases altering their normal procedures to meet the extraordinary circumstances. These adaptive and transformative capacities are some of the hallmarks that define a resilient healthcare system [40]. As the USVI faces an annual hurricane season that is forecasted to intensify over time, it will be necessary to approach hurricane preparedness with intentionality in increasing resiliency and responding to lessons learned from prior hurricanes.

Within the original conceptual framework, the maternity system is one component located within the larger societal/system level. However, there were few reflections by participants on underlying political, social, or economic structures. Hurricanes, like other “natural” disasters, are hazards shaped by government capacity, socioeconomic inequality, and other vulnerabilities. For example, in the case of Puerto Rico, another US territory with a similar colonial trajectory, the social, economic and health impacts of hurricane exposure are tied to historical and ongoing colonialism, the decentralization of the hurricane preparedness and response efforts, and pre-existing socioeconomic disparities [41]. However, while participants were asked to comment on how living in a US territory may have impacted their experiences, reflections on this historical trajectory did not emerge. Therefore, the analysis of system-related factors focused on the maternity system- the area of primary concern for the participants.

### Implications

#### Policy implications

Given the vulnerabilities specific to pregnant women in hurricane prone areas, our findings suggest that institutions at all levels of the maternity system, including private healthcare practices, clinics, hospitals, and the local department of health (DOH) should initiate collaborative hurricane preparedness and response plans [42, 43]. It is common for larger institutions such as hospitals and the DOH to have hurricane preparedness policies in place but less so for smaller clinics and private healthcare practices. However, participants in this study reported experiencing challenges specific to the ambulatory care setting such as communicating with their healthcare provider, accessing medical records, and maintaining continuous prenatal care. These findings indicate that leadership in clinics and private healthcare practices should think critically about the anticipated hurricane impacts on care and create policies and procedures to address these impacts. The DOH, as a coordinating and regulatory body, should require that these institutions develop policies that are specific to their setting and in alignment with governmental and hospital policies. Additionally, members of the maternity system should also engage in simulation trainings [44]. Ensuring continuous readiness at all levels of care is critical to maintaining quality maternity care after hurricanes.

#### Practice implications

Hospitals, healthcare practices, and healthcare providers can adapt existing guidance to develop hurricane preparedness plans and to facilitate patient hurricane emergency readiness [45–48]. These plans should ensure post-hurricane access to electronic medical records. Additionally, developing a post-hurricane communication plan is essential given the possibility of nonfunctioning landline or cell phone lines. If cell phone lines are operating, a dedicated emergency cell phone or text line can facilitate the communication of updated office hours, new office locations, and medical evacuation. Providers should encourage patients to develop personal and household hurricane emergency plans that include considerations for safeguarding food, water, medicines, radios, and other supplies; finding a safe place to stay, giving birth in place [48]; and preventing injury. Pre-emptive discussions regarding medical evacuation procedures with anyone who is experiencing a high-risk pregnancy are critical. Healthcare providers should be prepared to manage and treat perinatal mood disorders, utilize psychological first aid to prevent mental health morbidity in the immediate hurricane aftermath, and maintain referral lists for psychiatric care [50]. All plans should be clearly communicated to patients prior to the start of hurricane season and reiterated as needed.

## Limitations

This study had several limitations. First, the interviews were conducted 22–24 months after the hurricanes and recall bias can be a concern. However, studies show that women are capable of remembering specific details from their pregnancy and birth many years later [51, 52]. Thus, recall bias was likely at least partially mitigated given the interview subject. Second, our sample consisted predominantly of individuals who stayed on the island for birth. Thus, the results may not adequately reflect the experience of women who left the island to give birth and are not fully generalizable to all women who were pregnant and exposed to the hurricanes. These two groups may differ significantly in terms of variables such as access to economic resources and pregnancy risk status. Therefore, future research should specifically address the experience of pregnant women who left the island after the hurricanes. Third, the sample did not include nurses, midwives, physicians, labor and birth staff, or representatives from the DOH. Narratives from these professionals could be particularly helpful in understanding the sequence of events prior to and after the Hurricanes, the rationale for hurricane preparedness and response policies, and the execution of these policies after Hurricanes Irma and Maria.

## Conclusion

This study explored experiences of risk and resilience among women who were pregnant during and after Hurricanes Irma and Maria in the USVI. Our findings provide qualitative evidence about how maternal exposure to hurricanes and the recovery period poses risks to prenatal nutrition status, personal safety, and the continuity of high-quality comprehensive maternity care. Despite exposure to these risks, women are able to utilize internal strategies and call on household and community sources of support to embolden resilience. The maternity system also persisted and utilized creative approaches to adapt to the challenges posed by the hurricanes and maintain critical maternal health services. As the USVI prepares for increasingly severe and frequent hurricanes, the findings from this study highlight the need for public-private partnerships, government leadership in developing and communicating hurricane preparedness and response policies and plans, and targeted mental and maternal health pre- and post-hurricane guidance and interventions to ensure that the short- and long-term needs of pregnant women experiencing hurricanes are met.

## Supplementary Information

**Additional file 1.**

## Data Availability

The datasets used and analyzed during the current study are available from the corresponding author on reasonable request.
