# Supplementary material for: Experiences of pregnant women exposed to Hurricanes Irma and Maria in the US Virgin Islands: a qualitative study
Source: BMC Pregnancy Childbirth. 2022 Dec 17;22:947. doi: 10.1186/s12884-022-05232-7 (PMC9759877; doi:10.1186/s12884-022-05232-7)
Supplement: Supplementary file 1 — Additional file 1. [file 12884_2022_5232_MOESM1_ESM.docx]

## Supplementary Material: Codebook

| 1 Fear of another hurricane |
| --- |
| 2 Overwhelm |
| 3 Speaking Up |
| 4 Resilience |
| 4.1 Maternity System Resilience |
| 4.2 Growth |
| 4.3 Bond |
| 4.4 Coping |
| 4.4.1 Getting on with it |
| 4.5 Community Resilience |
| 5 Danger or Emergency |
| 6 Good Quote |
| 7 Past Hurricane Experience |
| 8 Impact on Family/Friends/Relationships |
| 9 Competing Priorities |
| 10 Recommendations |
| 10.1 Support |
| 10.2 Future Coping Mechanisms |
| 10.3 Information |
| 10.4 Leave |
| 10.5 Pre Hurricane Guidance for Pregnant Women |
| 11 Impact of Living in a US Territory |
| 11.1 Lack of publicity to USVI |
| 12 Pregnancy and Birth |
| 12.1 Left Island for Birth/Medical Care |
| 12.2 Complications |
| 12.3 Zika |
| 12.4 Stay or Leave |
| 12.5 Experience at Hospital |
| 12.5.1 Opinion of Care |
| 12.5.2 Low Supplies |
| 12.5.3 Hospital Damage/Condemnation |
| 12.6 Birth |
| 13 Post-Hurricane |
| 13.1 Damage to Home |
| 13.2 Taking Stock |
| 13.3 Employment |
| 13.3.1 Not Helpful |
| 13.3.2 Helpful |
| 13.4 Left Island Temporarily |
| 13.5 Pediatric Care |
| 13.6 Lack of Support from Friends/family |
| 13.7 Impact on Hours and Processes |
| 13.8 Postpartum Care |
| 13.9 Caring for Child |
| 13.10 Poor Communication |
| 13.11 Displacement |
| 13.12 Support from Fam and Friends |
| 13.13 Financial |
| 13.14 Post-Hurricane Food & Water |
| 13.15 Lack of Electricity |
| 13.16 Resources |
| 13.16.1 Lack of items |
| 13.16.2 Unable to Benefit |
| 13.16.3 WIC/Other Social Services |
| 13.16.4 Disaster Resources |
| 13.17 Impact on Health |
| 13.17.1 Sought mental health therapy |
| 13.17.2 Stress |
| 13.17.2.1 In Utero Stress |
| 13.17.3 Sadness or Depression |
| 13.18 Prenatal Care Post-Hurricane |
| 13.18.1 Contacting OB Office/Provider |
| 13.18.2 Delay/Interruption in PNC |
| 13.18.3 Damage to OB Provider Office |
| 13.19 Impact of Hurricane on Pregnancy Perception |
| 13.20 What Made It Hard |
| 14 Pre-Hurricane |
| 14.1 Wasn't going to be as bad |
| 14.2 Plans for Birth |
| 14.3 Finding a Safe Place to Stay |
| 14.4 Expectations |
| 14.5 Preparing |
| 14.6 Pre-Hurricane Concerns |
| 15 Lines |
| 16 Hurricane Irma-STX or Maria-STT |
| 17 Night of Hurricane |
| 17.1 Damage to House |

**1 Fear of another hurricane**

**2 Overwhelm**

Narratives by participants that describe strong feelings of being overwhelmed by specific aspects of the hurricane recovery. They may indicate that a particular stressor or circumstance was too great to manage effectively. This may include descriptions of purposeful inaction or avoidance as a result of the feelings of overwhelm.

**3 Speaking Up**

Ways in which the participant stated that they advocated for themselves, the pregnancy or their child.

**4 Resilience**

Descriptions of resilience that include exemplar quotes that reflect: “the capacity of a dynamic system to adapt successfully to disturbances that threaten the viability, the function, or the development of that system.”

**4.1 Resilience\Maternity System Resilience**

Descriptions of the ways in which the healthcare system responded to the impacts of the hurricane in ways that allowed it to meet the needs of the pregnant population. This may include actions by hospitals, clinics/health care centers, or individual providers.

**4.2 Resilience\Growth**

Participant perceptions that they experienced personal growth as a result of what they experienced with the hurricanes.

**4.3 Resilience\Bond**

Descriptions of bonds or shared experiences among individuals or groups that experienced the hurricane.

**4.4 Resilience\Coping**

Descriptions of coping (attempts to deal with difficulties or overcome challenges).

**4.4.1 Resilience\Coping\Getting on with it**

A specific method of coping employed by participants that is task-oriented. rather than emotion-oriented.

**4.5 Resilience\Community Resilience**

Descriptions of resilience within the community. These may include descriptions of the ways that the community absorbed the disruption from the hurricane but was able to adapt to normality or to achieve collective wellbeing.

**5 Danger or Emergency**

Descriptions about instances in which the participant may have been in danger or experienced an emergency.

**6 Good Quote**

Quotes that are particularly interesting or salient.

**7 Past Hurricane Experience**

Mentions of experiences with past hurricanes (prior to the 2017 hurricanes).

**8 Impact on Family/Friends/Relationships**

Descriptions of how family, friends or relationships were impacted by the events or consequences of the hurricanes.

**9 Competing Priorities**

Descriptions of participants having to consider multiple needs and/or decide which need to prioritize.

**10 Recommendations**

Participant recommendations for improving the pre-hurricane preparedness or post-hurricane experience for pregnant women and their infants.

**10.1 Recommendations\Support**

Participant recommended that pregnant women receive additional support when dealing with hurricanes.

**10.2 Recommendations\Future Coping Mechanisms**

Participant recommended specific mechanisms for coping with pregnancy and hurricanes.

**10.3 Recommendations\Information**

Participant recommended additional information.

**10.4 Recommendations\Leave**

Participant recommended that pregnant women leave the island in the face of future hurricanes.

**10.5 Recommendations\Pre-Hurricane Guidance for Pregnant Women**

Participant recommended that pregnant women receive specific pre-hurricane guidance.

**11 Impact of Living in a US Territory**

Participants describe what impact living in a US territory may have had on their experience.

**11.1 Impact of Living in a US Territory\Lack of publicity to USVI**

Participant mentions that there was a lack of publicity on the USVI after the hurricanes.

**12 Pregnancy and Birth**

Participant descriptions of their pregnancy or birth.

**12.1 Pregnancy and Birth\Left Island for Birth/Medical Care**

Description of leaving the island for medical care during their pregnancy or specifically for birth.

**12.2 Pregnancy and Birth\Complications**

Mentions of specific complications that the participant experienced in their pregnancy or birth pre- or post-hurricane.

**12.3 Pregnancy and Birth\Zika**

Participant mentions Zika. This may include mentions of concerns of Zika virus infection, testing or test results, or preventing Zika virus infection.

**12.4 Pregnancy and Birth\Stay or Leave**

Descriptions of thoughts around staying or leaving the island before or after the Hurricanes. This may include recounting of conversations with family and friends, internal thoughts, and the decision-making process.

**12.5 Pregnancy and Birth\Experience at Hospital**

Descriptions of giving birth (or maternity surveillance) at the hospital.

**12.5.1 Pregnancy and Birth\Experience at Hospital\Opinion of Care**

Participant’s personal opinion of the quality of care that they received.

**12.5.2 Pregnancy and Birth\Experience at Hospital\Low Supplies**

Mention of there not being enough of a particular supply or product at the hospital.

**12.5.3 Pregnancy and Birth\Experience at Hospital\Hospital Damage/Condemnation**

Mention of damage to the hospital or mention of the hospital being condemned.

**12.6 Pregnancy and Birth\Birth**

Participant description of the events of their labor, birth and postpartum.

**13 Post-Hurricane**

Descriptions of events of conditions after the hurricanes.

**13.1 Post-Hurricane\Damage to Home**

**13.2 Post-Hurricane\Taking Stock**

Participant descriptions of what they saw or experienced the first day after the hurricane (landscape, damage, curfew, and more).

**13.3 Post-Hurricane\Employment**

Descriptions surrounding their employment status and situation.

**13.3.1 Post-Hurricane\Employment\Not Helpful**

Participant recounts ways in which their employer, fellow employees, or supervisor were not helpful in providing information, resources or support.

**13.3.2 Post-Hurricane\Employment\Helpful**

Participant recounts ways in which their employer, fellow employees, or supervisor were helpful in providing information, resources or support.

**13.4 Post-Hurricane\Left Island Temporarily**

Descriptions of leaving the island temporarily for non-medical reasons.

**13.5 Post-Hurricane\Pediatric Care**

Descriptions of securing or receiving pediatric care for their infant.

**13.6 Post-Hurricane\Lack of Support from Friends/family**

Descriptions of not having support from family and friends after the hurricanes.

**13.7 Post-Hurricane\Impact on Hours and Processes**

Descriptions of ways in which the hurricanes impacted business hours and processes on the island.

**13.8 Post-Hurricane\Postpartum Care**

Descriptions around receiving postpartum care after the hurricanes.

**13.9 Post-Hurricane\Caring for Child**

Descriptions around caring for their infant after the hurricanes.

**13.10 Post-Hurricane\Poor Communication**

Descriptions of poor communication from businesses or the government after the hurricanes.

**13.11 Post-Hurricane\Displacement**

Descriptions of being displaced from their home.

**13.12 Post-Hurricane\Support from Fam and Friends**

Descriptions of the ways in which family and friends provided support after the hurricanes.

**13.13 Post-Hurricane\Financial**

Descriptions of ways in which the hurricanes impacted the finances of the participant.

**13.14 Post-Hurricane\Post-Hurricane Food & Water**

Descriptions of hurricane impact on access to clean water and food. This may also include descriptions of the ways in which participants secured these items.

**13.15 Post-Hurricane\Lack of Electricity**

Description of the hurricane’s impact on electricity and descriptions of what it was like to live without electricity, or concerns around caring for newborn without electricity.

**13.16 Post-Hurricane\Resources**

Descriptions of resources that participants either needed, took advantage of, or wanted to take advantage of after the hurricanes.

**13.16.1 Post-Hurricane\Resources\Lack of items**

Specific descriptions of items that the participant needed related to pregnancy and infant care.

**13.16.2 Post-Hurricane\Resources\Unable to Benefit**

Descriptions of the participant’s inability to benefit from a desired resource.

**13.16.3 Post-Hurricane\Resources\WIC/Other Social Services**

Description of use of WIC or other social services after the hurricane.

**13.16.4 Post-Hurricane\Resources\Disaster Resources**

Description of disaster-specific resources such as D-SNAP, FEMA issued funds for reimbursement for damaged household items, or tangible resources to aid with securing their house or roof.

**13.17 Post-Hurricane\Impact on Health**

Mention of the impacts the hurricanes had on their health.

**13.17.1 Post-Hurricane\Impact on Health\Sought mental health therapy**

Mention of seeking mental health therapy or counseling after the hurricanes.

**13.17.2 Post-Hurricane\Impact on Health\Stress**

Descriptions of the impact of hurricane-related stress on the participant.

**13.17.2.1 Post-Hurricane\Impact on Health\Stress\In Utero Stress**

Mention by the participant that they were concerned that the stress associated with the hurricane may impact the developing fetus. Also includes mention on specific attempts to reduce or control stress or emotions so as not to disturb the fetus.

**13.17.3 Post-Hurricane\Impact on Health\Sadness or Depression**

Descriptions of sadness or depression that the participant attributes to their experience after the hurricanes.

**13.18 Post-Hurricane\Prenatal Care Post-Hurricane**

Descriptions about receiving prenatal care post-hurricane.

**13.18.1 Post-Hurricane\Prenatal Care Post-Hurricane\Contacting OB Office/Provider**

Participant descriptions of how they contacted their OB provider to make appointments after the hurricane.

**13.18.2 Post-Hurricane\Prenatal Care Post-Hurricane\Delay/Interruption in PNC**

Descriptions of delays or interrupted prenatal care.

**13.18.3 Post-Hurricane\Prenatal Care Post-Hurricane\Damage to OB Provider Office**

Descriptions of damaged OB provider offices.

**13.19 Post-Hurricane\Impact of Hurricane on Pregnancy Perception**

Mention of how the hurricane impacted their perception of their pregnancy.

**13.20 Post-Hurricane\What Made It Hard**

Descriptions of “what made it hard” to be pregnant and deal with a hurricane simultaneously.

**14 Pre-Hurricane**

Descriptions of life, plans, expectations or perceptions the participant had prior to the hurricanes.

**14.1 Pre-Hurricane\Wasn't going to be as bad**

**14.2 Pre-Hurricane\Plans for Birth**

Description of pre-hurricane plan for birth.

**14.3 Pre-Hurricane\Finding a Safe Place to Stay**

Description of finding a safe place to stay for the night of the hurricane.

**14.4 Pre-Hurricane\Expectations**

Description of what the participant thought or expected pregnancy and birth to be like prior to the hurricanes.

**14.5 Pre-Hurricane\Preparing**

Descriptions of disaster preparations made prior to hurricane.

**14.6 Pre-Hurricane\Pre-Hurricane Concerns**

Descriptions of concerns that participant had about the storms in the days leading up to the hurricanes.

**15 Lines**

Descriptions of standing in line for resources/goods/services after the hurricane.

**16 Hurricane Irma-STX or Maria-STT**

Mentions of experiences with Hurricane Irma (only if they were on STX) or Hurricane Maria (only if they were on STT).

**17 Night of Hurricane**

Descriptions of the night of the hurricane(s).

**17.1 Night of Hurricane\Damage to House**
